# Supplementary material for: Identification of photoperiod-induced specific miRNAs in the adrenal glands of Sunite sheep (Ovis aries)
Source: Front Vet Sci. 2022 Jul 22;9:888207. doi: 10.3389/fvets.2022.888207 (PMC9354845; doi:10.3389/fvets.2022.888207)
Supplement: Supplementary file 1 [file Data_Sheet_1.ZIP › Supplementary file/miRDeep2 file.zip!/miRDeep2 file/Novel_Structure/SPLP42A2/Novel_398.pdf]

Provisional ID	:	NC_019479.2_37417
Score total	:	2.5
Score for star read(s)	:	-1.3
Score for read counts	:	0
Score for mfe	:	2.2
Score for randfold	:	1.6
Score for cons. seed	:	
Total read count	:	265
Mature read count	:	265
Loop read count	:	0
Star read count	:	0

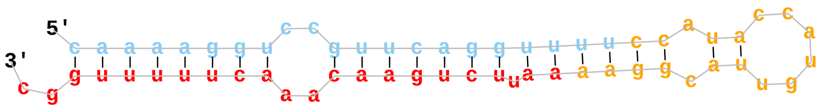

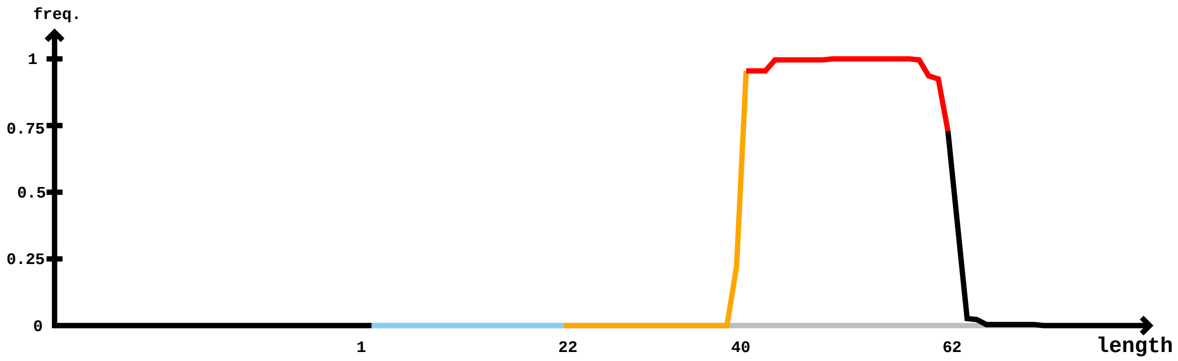

Star   Mature

```
5'- cuuuugagagccuguuaaauacuggguuggccaaaagguccguucagguuuuccauaccauguuacggaaaauucugaacaaacuuuuuggccaacccaauauuuaccagug   -3'    exp
    ...........(((.((((((.(((((((((((((((((..(((((((((((((.((......)).)))))).)))))))..))))))))))))))))).)))))).)))..    reads      mm          sample
    .....................................................................aaaAucugaacaaacuuuu........................    1          1           seq
    .....................................................................aaaAucugaacaaacuuuuu.......................    15         1           seq
    .....................................................................aaaAucugaacaaacuuuuugg.....................    39         1           seq
    .....................................................................aaaAucugaacaaacuuuuuggc....................    4          1           seq
    ......................................................................aaAucugaacaaacuuuuu.......................    1          1           seq
    ......................................................................aaAucugaacaaacuuuuug......................    3          1           seq
    ......................................................................aaAucugaacaaacuuuuugg.....................    13         1           seq
    ......................................................................aauucugaacaaacuuuuuggc....................    1          0           seq
    ......................................................................aaAucugaacaaacuuuuuggc....................    177        1           seq
    .........................................................................ucugaGcaaacuuuuuggc....................    1          1           seq
    .........................................................................ucugaacaaaAuuuuuggcc...................    1          1           seq
    .........................................................................ucugaacaaacuuuuuggcA...................    3          1           seq
    .........................................................................ucugaacaaacuuuuuggcAa..................    1          1           seq
    .........................................................................ucugaacaaacuuuuuggccaC.................    5          1           seq
    ...............................................................................caaacuuuuuggccaacccaUu...........    1          1           seq
```
